# Systematic Review and Meta-Analysis on the Infection Rates of Schistosome Transmitting Snails in Southern Africa

**DOI:** 10.3390/tropicalmed7050072

**Published:** 2022-05-13

**Authors:** Onyekachi Esther Nwoko, Chester Kalinda, Moses John Chimbari

**Affiliations:** 1 Discipline of Public Health Medicine, School of Nursing and Public Health, College of Health Sciences, Howard Campus, University of KwaZulu-Natal, Durban 4000, South Africa; ckalinda@gmail.com (C.K.); chimbari@ukzn.ac.za (M.J.C.); 2 Bill and Joyce Cummings Institute of Global Health, University of Global Health Equity (UGHE), P.O. Box 6955, Kigali 20093, Rwanda; 3 Institute of Global Health Equity Research (IGHER), University of Global Health Equity (UGHE), P.O. Box 6955, Kigali 20093, Rwanda; 4 Department of Behavioural Science, Medical and Health Sciences, Great Zimbabwe University, Masvingo P.O. Box 1235, Zimbabwe

**Keywords:** *Bulinus* spp., *Biomphalaria* spp., schistosomiasis, intermediate host snails, infection rates

## Abstract

Efforts to interrupt and eliminate schistosomiasis as a public health problem have increased in several Southern African countries. A systematic review was carried out on the infection rates of snails that cause schistosomiasis in humans. The searches were conducted in PubMed, Web of Science, and Scopus databases, using the PRISMA guidelines from inception to 24 February 2022. The study quality was assessed by using the Joanna Briggs Institute prevalence critical appraisal checklist. Pooled infection rates were estimated by using an inverse variance heterogeneity model, while heterogeneity was determined by using Cochran’s Q test and Higgins i^2^ statistics. A total of 572 articles were screened, but only 28 studies were eligible for inclusion based on predetermined criteria. In the selected studies, 82,471 *Bulinus* spp. and 16,784 *Biomphalaria* spp. snails were screened for cercariae. The pooled infectivity of schistosome intermediate host snails, *Biomphalaria* spp., and *Bulinus* spp. were 1%, 2%, and 1%, respectively. Snail infection rates were higher in the 1900s compared to the 2000s. A Luis Furuya–Kanamori index of 3.16 indicated publication bias, and a high level of heterogeneity was observed. Although snail infectivity in Southern Africa is relatively low, it falls within the interval of common snail infection rates, thus indicating the need for suitable snail control programs that could interrupt transmission and achieve elimination.

## 1. Introduction

Schistosomiasis is a neglected tropical disease (NTD) that mainly affects poor and marginalized communities in sub-Saharan Africa [1,2]. The two major forms of schistosomiasis affecting humans in sub-Saharan Africa are intestinal and urogenital schistosomiasis. Intestinal schistosomiasis is caused by blood fluke trematodes *Schistosoma mansoni*, *S. intercalatum*, and *S. guineensis*, while urogenital schistosomiasis is caused by *S. haematobium* [3]. The transmission cycle involves the release of eggs from infected humans into freshwater bodies through faeces or urine. These eggs hatch and release miracidia which penetrate suitable intermediate host snails. *S. mansoni* penetrates *Biomphalaria* spp. snails, *S. haematobium* penetrates *Bulinus* spp. snails., and *S. mansoni* could penetrate *Bulinus* spp. snails sometimes. Once miracidia infects the suitable snails, sporocysts develop in the snails, which then release cercariae into the water after the prepatent period [4]. Snails can shed hundreds of cercariae daily, ranging around 200 for *S. haematobium* and 250 to 600 for *S. mansoni* [5,6].

Substantial progress has been made over the years to prevent and control schistosomiasis by implementing large-scale periodic treatment with praziquantel. However, this has not completely interrupted schistosomiasis transmission [7]. Suggestions have been made on the need to adopt integrated control strategies, including preventive chemotherapy with praziquantel, intermediate host snail management, and improved water and sanitation. Following the adoption of the WHA70.16 on the Global Vector Control Response [8], there is a need for updated data on the infectivity of intermediate host snails (IHS) of schistosomiasis to justify investment in snail control and develop more effective prevention and control programs.

Many malacological studies on the infectivity of IHS in countries in Southern Africa have been conducted yet there has not been any single estimate of the infectivity of IHS for the entire Southern African countries. Such information would be essential in aiding policymakers working on the prevention and control of schistosomiasis. Hailegebriel et al. [9] reported a 6% prevalence of *S. mansoni* and *S. haematobium* in snail intermediate hosts in Africa. The study further suggested an increase in the pooled prevalence of schistosome cercaria in recent years among freshwater snails. However, only one study from the entire Southern Africa region was included, thus, potentially underestimating the potential risks of infections in this region, which has reported high schistosomiasis infections and snail abundance in several areas [9]. Hence, this study was designed to conduct a systematic review and meta-analysis on a micro-geographical scale in Southern African countries to estimate the infectivity of IHS. A clear understanding of IHS infectivity is pivotal for effective planning of targeted disease control and sustainable strategies to interrupt schistosomiasis transmission.

## 2. Materials and Methods

### 2.1. Search Strategies and Inclusion Criteria

A comprehensive literature search of published articles on the infectivity of intermediate host snails that transmit the parasite that causes schistosomiasis in humans in Southern Africa was systematically conducted in PubMed, Web of Science, and Scopus databases from inception to 24 February 2022. The following search terms were used: “schistosome intermediate host”, “intermediate host snails”, “snail intermediate host”, “intermediate host”, “freshwater snails”, “freshwater snail host”, “snail vector”, “malacology survey”, “*Biomphalaria*”, “*Bulinus*”, “Bulinid”, “infection”, “infection rate”, “intensity”, “prevalence”, “incidence”, “schistosomiasis”, “bilharzia”, “bilharziasis”, “*Schistosoma mansoni*”, “*S. haematobium*”, “Schistosoma”, “Angola”, “Botswana”, “Lesotho”, “Malawi”, “Mozambique”, “Namibia”, “South Africa”, “Swaziland”, “Eswatini”, “Zambia”, and “Zimbabwe” (Supplementary Materials File S1). Search terms were combined by using the AND/OR Boolean operators. Our search was limited to peer-reviewed articles published in the English language. Relevant articles were also identified from the reference list of already identified articles. The Preferred Reporting Items for Systematic Reviews and Meta-analyses (PRISMA) guideline was used for the paper selection process [10].

The inclusion criteria for all articles were as follows: (a) studies reporting data from any Southern African country, (b) studies reporting data on human schistosomiasis intermediate host snails (*Biomphalaria* spp. and *Bulinus* spp.) to species level, (c) studies reporting the number of examined and infected snails with human schistosomes, (d) studies that mentioned the diagnostic used in detecting infected snails, and (e) studies that reported infection in snails that had been sampled from the field and not laboratory infected snails. Studies without full texts, review articles, and meta-analysis were excluded.

### 2.2. Data Extraction and Quality Appraisal

The data extraction format from the reviewed papers included the first author’s name, year of publication, study country, snail species, number of snails (collected, examined, and infected), and diagnostics used in detecting schistosome infection. The quality of all studies included was assessed by using the 10 quality-control items described by the Joanna Briggs Institute Prevalence Critical Appraisal [11]. A score of 1 was given for each item fulfilled, while 0 was given for each unfulfilled item. An aggregate of all the scores was generated and converted into an index. Based on the quality indices generated, studies were classified as having low (0.0–0.3), moderate (0.4–0.6), or high (0.7–1.0) quality (Supplementary Materials File S2).

### 2.3. Statistical Analysis

An inverse variance heterogeneity (IVhet) model in MetaXL version 5.3 (meta-analysis add-in tool in Microsoft Excel) was used to obtain the pooled prevalence estimates from the eligible studies. The IVhet model was used because, irrespective of heterogeneity, the confidence interval coverage remains close to the nominal level compared to the fixed-effect and random-effect models, where the confidence interval drops significantly [12,13,14]. Forest plots were generated to show the estimated prevalence and their 95% confidence interval. The level of heterogeneity between studies was measured by using Cochran’s Q statistic, and Higgin’s inconsistency statistic ($i^{2}$) was used to estimate the proportion of variability between studies. Higgins’s $i^{2}$ can be considered to show strong homogeneity, medium heterogeneity, and high heterogeneity when it has a value less than 25%, 50%, and 75%, respectively [15]. Publication bias was assessed by using the Luis Furuya–Kanamori (LFK) index of the Doi plot. The level of publication bias depended on the magnitude of the LFK index. An LFK value within the range of ‘±1’ was considered as ‘symmetrical’ and classified as the absence of publication bias, an LFK value within the range of ‘±2’ was considered as minor asymmetry with slight publication bias, and an LFK value outside the range of ‘±2’ was considered as major asymmetry and high publication bias. Furthermore, subgroup analysis was carried out by stratifying our data by snail species and the countries where the studies were conducted to potentially explain the observed heterogeneity [16,17].

## 3. Results

### 3.1. Search Results

**Figure 1:**
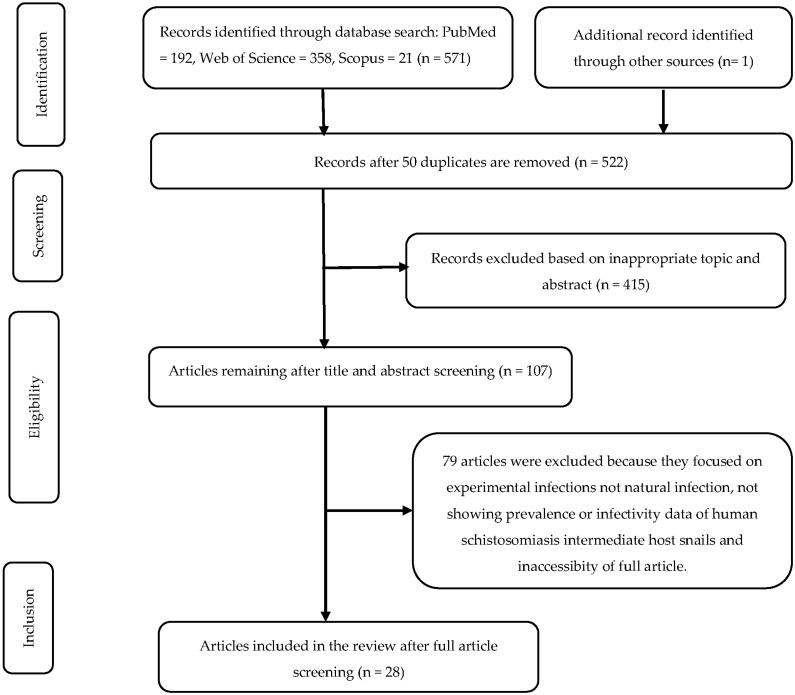
PRISMA flow diagram of the studies included in the meta-analysis.

A total of 572 articles were identified, and 50 duplicated articles were removed. The remaining 522 records were screened by using the titles and abstracts, and 415 were excluded. One hundred and seven articles were then evaluated according to eligibility criteria. Seventy-nine articles were also excluded. Finally, 28 articles were included in the study, as they passed the eligibility criteria and quality assessment (Figure 1). The studies included in this review ranged from 1954 to 2021.

### 3.2. Study Characteristics and PPE Analysis

The twenty-eight (28) eligible studies included in the review were conducted in seven Southern Africa countries; 3.6% (*n* = 1) [18] were from Angola, 7.1% (*n* = 2) [19,20] were from Botswana, 17.9.5% (*n* = 5) [21,22,23,24,25] were from Malawi, 3.6% (*n* = 1) [26] were from Mozambique, 17.9% (*n* = 5) [27,28,29,30,31] were from South Africa, 3.6% (*n* = 1) [32] were from Zambia, and 46.4% [33,34,35,36,37,38,39,40,41,42,43,44,45] (*n* = 13) were from Zimbabwe. Of the 28 studies, 6 studies reported on the infectivity rate in *Bulinus* spp., 5 studies reported on *Biomphalaria* spp., and 14 studies reported on both *Bulinus* spp. and *Biomphalaria* spp. All the studies used the cercarial shedding diagnostic to detect schistosome infections in the intermediate host snails. Furthermore, 10 studies were cross-sectional, and 18 studies were longitudinal (Table 1). Moreover, more snails were found in studies with a long study duration.

**Figure 2:**
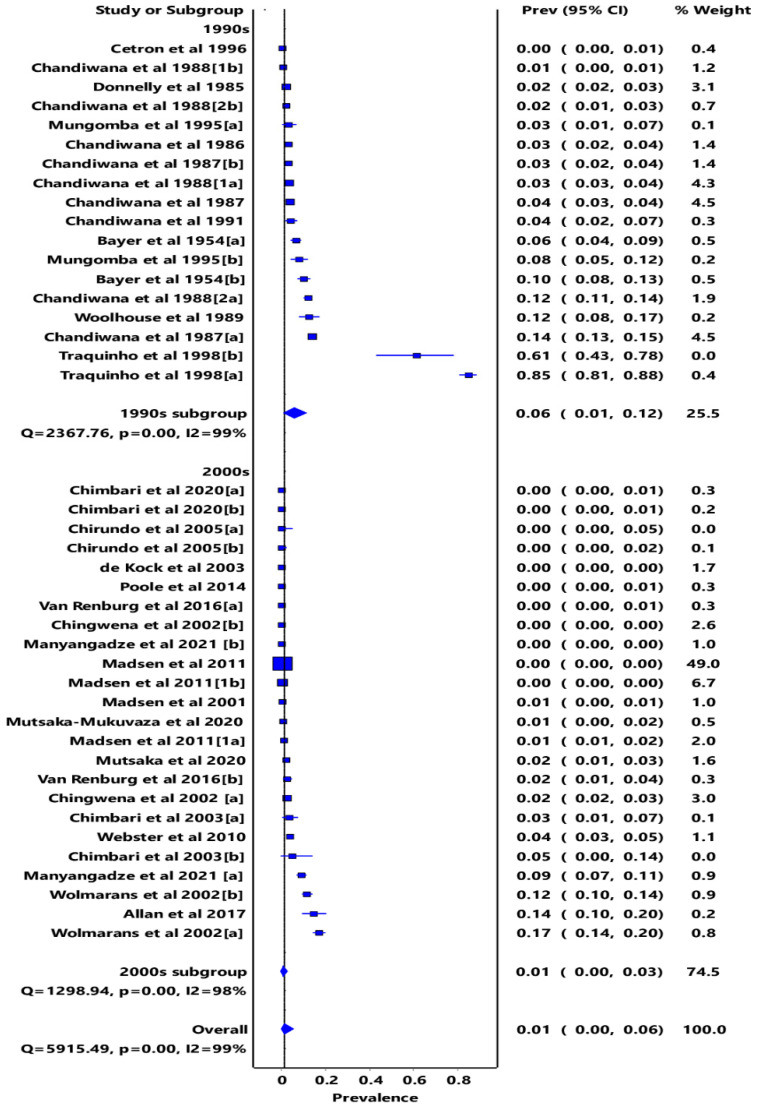
Forest plot of subgrouped PPE analysis of infectivity in the 1900s and 2000s [18,19,20,21,22,23,24,25,26,27,28,29,30,31,32,33,34,35,36,37,38,39,40,41,42,43,44,45]. [a] and [b] represents Bulinus spp. and *Biomphalaria* spp. respectively when both species are of interest to the author(s). [1a] and [1b] represents *Bulinus* spp. and *Biomphalaria* spp. respectively when both species are of interest to the author(s) and is the first of two articles published in the same year by the same authors. [2a] and [2b] represents *Bulinus* spp. and *Biomphalaria* spp. respectively when both species are of interest to the author(s) and is the second article published in the same year.

The overall pooled prevalence estimate (PPE) of infectivity was 1% (95% CI: 0.00–0.06), with a high degree of heterogeneity ($i^{2}$ = 99%, *p* < 0.01) (Figure 2). The years of the studies included in the review were categorized into two groups, namely; the 1990s and 2000s, to assess the trends of snail infection rates in intermediate host snails between the periods. The pooled snail infectivity was 6% (95% CI: 0.01–0.12) in the 1990s and 1% (95% CI: 0.00–0.03) in the 2000s (Figure 2). The highest pooled prevalence of schistosome cercariae was obtained among freshwater snails from Mozambique (83%; 95% CI, 0.53–1.00), followed by Angola (14%; 95% CI, 0.10–0.20), Zambia (83%; 95% CI; 0.02–0.12), Zimbabwe (83%; 95% CI, 0.02–0.07), South Africa (83%; 95% CI; 0.00–0.09), Botswana (83%; 95% CI, 0.00–0.02), and Malawi (83%; 95% CI, 0.00–0.01) (Supplementary Figure S1). The subgroup analysis stratified by snail species showed that the rate of infectivity in *Bulinus* spp. was 1% (95% CI: 0.00–0.07), while in *Biomphalaria* spp., it was 2% (95% CI: 0.00–0.04) (Supplementary Figure S2). A high level of heterogeneity was observed ($i^{2}$ > 90%), and this could not be reduced through subgroup analysis by intermediate host snail species. This could be due to the differences in the seasons the data were collected or the study designs [46]. A significant publication bias was observed both from the funnel and doi plots, as shown by the LFK index of 3.16, which indicates major asymmetry (Supplementary Figures S3 and S4).

Table 1 presents information extracted from the 28 eligible articles that included citation name, study duration, sample size, positive, infection rate, snail species, country, method of diagnosis and sampling type.

## 4. Discussion

In sub-Saharan Africa, schistosomiasis is one of the leading infectious diseases of public health importance coming after malaria [47,48,49]. In this meta-analysis, we analyzed studies on the infectivity of schistosome IHS in Southern Africa. Our findings show that the overall pooled prevalence of schistosome IHS in Southern Africa is low. This corroborates with previous studies which concluded that IHS infectivity can be as low as 1 to 2%, even in areas with a high prevalence of humans infected with schistosomiasis [50,51,52,53]. In Kisumu city, Western Kenya, the proportion of snails shedding schistosome cercariae was 1.8% despite the prevalence of schistosomiasis among school children being 21% and 3.6% for *S. mansoni* and *S. haematobium*, respectively [54,55]. Furthermore, a study performed in Msambweni, along the Kenyan coast, reported a snail infectivity rate of 1.2% [56], while the prevalence of *S. haematobium* among residents was 32.4% [57]. In the Lake Victoria basin in Western Kenya, snail infectivity was reported as 1.04% [58], whereas a high prevalence of *S. mansoni* of 60.5% was recorded among schoolchildren [59]. In Senegal, Catalano et al. [60] reported that 12.8% of school-aged children had *S. mansoni* infections, while the recorded snail infectivity was 2.2%. The prevalence of *S. mansoni* and *S. haematobium* in Toho-Todougha, Benin, was 74.3% and 57.1%, respectively, but the infection rate of *B. pfeifferi* was 0.56%, and the infectivity of *B. forskalii* and *B. globosus* were both 0% [61]. In Unguja Island, Tanzania, *S. haematobium* prevalence in school-aged children was 16.8% and 2.3% snail infectivity [62]. Several factors may be responsible for the discrepancy between snail infectivity and schistosomiasis prevalence. They include the method used in detecting schistosome infection, the ability of a few snails to release thousands of cercariae in a day, with the peak time for shedding cercariae from 9:00 to 11:00 a.m., followed by a decline at 5:00–7:00 p.m. [31,63]. This diurnal pattern of snail shedding coincides with the time people have intense water contact.

In contrast to the above, there are studies with high schistosomiasis prevalence and high snail infectivity. In our review, Cabo Delgabo province in Northern Mozambique, one of the least developed areas in the country, had the highest snail infectivity, at 83% [26]. This could be attributed to the high endemicity of schistosomiasis in the country, where they recorded a schistosomiasis prevalence of 84.4% [26]. The Schistosomiasis Consortium for Operational Research and Evaluation (SCORE) in 2011 coordinated a five-year study that was implemented in various African countries [64]. The goal of the SCORE project was to research integrated strategies that might stop transmission and achieve elimination. Preventive chemotherapy by using praziquantel treatment in Cabe Delgabo, Mozambique, resulted in a significant reduction in the prevalence of *S. haematobium* infection from Year 1 to Year 5, where the average prevalence reduced from 60.5% to 38.8% [64]. Despite this achievement, data on snail infectivity were not updated, thus suggesting the need for more malacology surveys in Mozambique. SCORE also undertook both malacology and parasitology surveys over 4 years to determine *Biomphalaria* snail abundance in Mwanza, Tanzania. A decrease in shedding *Biomphalaria* abundance in Year 4 was observed, and this was attributed to the schistosomiasis treatment that was ongoing in the human populations [65].

The pooled prevalence of intermediate host snails decreased over time, from 6% in the 1900s to 1% in the 2000s. This might be attributed to the increased health education in raising awareness on how schistosomiasis is contracted, and it has led to new lifestyles, improved water and sanitation, ongoing snail control programs, and the effects of climate change.

*Biomphalaria* snails serve as intermediate hosts for *S. mansoni*, while *Bulinus* snails act as intermediate hosts for *S. haematobium* and *S. mattheei* in Southern Africa [66]. This review showed that *Bulinus* snails were more abundant (*n* = 82,471) compared to *Biomphalaria* snails (*n* = 16,784). This could be because *Bulinus* snails are better equipped to withstand extreme temperatures, swift currents, and prolonged droughts better than *Biomphalaria* snails [67,68]. This finding is in contrast with the results obtained by Kinanpara et al. [69] and Hailegebriel, Nibret, and Munshea [9], where *Biomphalaria* snails had a higher abundance. *Biomphalaria* snails, on the other hand, had higher infectivity (2%) compared to *Bulinus* snails (1%) in Southern Africa.

In all the studies reviewed, the cercarial shedding technique was used to detect schistosome infections. This is a common diagnostic method used in detecting infections because it is relatively affordable and easy to carry out [55]. However, the cercarial shedding technique is known to underestimate the true prevalence of infection in the intermediate host snail, due to its inability to detect prepatent infections, the aborted development of sporocysts, and the death of the snail after collection and before light exposure, as well as being time-consuming and labor-intensive [2,70]. In addition, further laboratory analysis is needed to identify the specific type of cercariae. Hence, the method of infection detection may be partly responsible for the low pooled prevalence in this meta-analysis. To overcome these limitations, different types of molecular diagnostics which detect *Schistosoma* DNA in intermediate host snails have been developed and can identify patent and prepatent infections [70,71,72,73,74]. Studies have shown a significant difference in the prevalence of IHS schistosome infection between cercariae shedding and molecular diagnostics. Infection rates of 0%, 3%, 1.56%, and 0% [52,71,75,76] were reported when the cercarial shedding technique was used. However, when nested polymerase chain reaction (PCR) was used, the reported infection rates were 2%, 3%, 39.6%, and 9.76% [52,71,75,76]. Furthermore, Sengupta et al. [77] reported that eDNA xenomonitoring detected schistosome presence at sites where cercarial shedding failed. However, due to the cost of the molecular diagnostic and the requirement of trained personnel to carry out molecular analysis, this approach is not commonly used. There is a need for the development of simple and field-friendly methods for the detection of *Schistosoma* in snails. This would provide a better picture of schistosomiasis in various countries in order to guide policymaking, prevention, and control [2].

A key limitation of our study and recommendation for additional work is the effect of seasonality on *Schistosoma* spp. infections in IHS. Of the 28 studies included in this review, only four studies [30,34,35,43] reported the effect of seasonality on *Schistosoma* infections. The studies showed an increased infection rate in the dry season compared to rainy seasons. However, only one out of the four studies reported the total number of snails collected in each season; hence, we could not ascertain the pooled prevalence per season. The seasonal pattern observed in the infection of *Biomphalaria* spp. and *Bulinus* spp. with mammalian schistosome cercariae where the prevalence of infection of snails with larval trematodes increased during the dry season and decreased during the rainy season is consistent with that observed in References [78,79].

## 5. Conclusions

The results from the review showed that 1% of *Bulinus* spp. and *Biomphalaria* spp. freshwater snails were infected. Although this overall pooled prevalence is low, the presence of an infected snail in a waterbody is evidence of schistosomiasis transmission and is of public health concern, as direct contact with the waterbody will lead to schistosomiasis infection. Furthermore, the cercarial shedding method used in detecting infection may have played a role in the low prevalence observed. Our results highlight the need for more malacology surveys using improved infectivity diagnostic methods in the Southern African region to enhance the detection of infection and integrated snail control strategies to interrupt schistosomiasis transmission.

## Figures and Tables

**Table 1:** Summary of eligible studies included in the meta-analysis.

Citation Name	Study Duration	Sample Size	Positive	Infection Rate (%)	Snail Species	Country	Method of Diagnosis	Sampling Type
Chimbari et al. (2003) [a] [33]	1 year	120	4	3.33	*B. globosus*	Zimbabwe	Cercarial shedding	Longitudinal
Chimbari et al. (2003) [33] [b]	1 year	42	2	4.76	*B. pfeifferi*	Zimbabwe	Cercarial shedding	Longitudinal
Chimbari et al. 2020 [a] [19]	3 years	303	0	0	*B. globosus*	Botswana	Cercarial shedding	Longitudinal
Chimbari et al. (2020) [b] [19]	3 years	199	0	0	*B. pfeifferi*	Botswana	Cercarial shedding	Longitudinal
Chandiwana et al. (1988) [1a] [38]	2 years	4237	142	3.35	*B. globosus*	Zimbabwe	Cercarial shedding	Longitudinal
Chandiwana et al. (1988) [1b] [38]	2 years	1163	9	0.77	*B. pfeifferi*	Zimbabwe	Cercarial shedding	Longitudinal
Mutsaka-Makuvaza et al. (2020) [43]	1 year	1542	30	1.95	*B. globosus*	Zimbabwe	Cercarial shedding	Longitudinal
Chirundo et al. (2005) [a] [41]	1 month	34	0	0	*B. globosus*	Zimbabwe	Cercarial shedding	Cross-sectional
Chirundo et al. (2005) [b] [41]	1 month	86	0	0	*B. pfeifferi*	Zimbabwe	Cercarial shedding	Cross-sectional
Allan et al. (2017) [18]	2 months	173	25	14.45	*B. globosus*	Angola	Cercarial shedding	Cross-sectional
Manyangadze et al. (2021) [a] [30]	1 year	861	77	8.94	*B. globosus*	South Africa	Cercarial shedding	Longitudinal
Manyangadze et al. (2021) [a] [30]	1 year	985	1	0.10	*B. pfeifferi*	South Africa	Cercarial shedding	Longitudinal
Chandiwana et al. (1986) [34]	2 years	1347	41	3.04	*B. pfeifferi*	Zimbabwe	Cercarial shedding	Longitudinal
Woolhouse et al. (1989) [45]	2 months	225	28	12.44	*B. globosus*	Zimbabwe	Cercarial shedding	Longitudinal
Traquinho et al. (1998) [a] [26]	2 months	407	345	84.77	*Bulinus* spp.	Mozambique	Cercarial shedding	Cross-sectional
Traquinho et al. (1998) [b] [26]	2 months	31	19	61.29	*Biomphalaria* spp.	Mozambique	Cercarial shedding	Cross-sectional
Bayer et al. (1954) [a] [27]	2 months	482	31	6.43	*Bulinus* spp.	South Africa	Cercarial shedding	Cross-sectional
Bayer et al. (1954) [b] [27]	2 months	520	53	10.19	*Biomphalaria* spp.	South Africa	Cercarial shedding	Cross-sectional
Cetron et al. (1996) [21]	2 months	370	1	0.27	*Bulinus* spp.	Malawi	Cercarial shedding	Cross-sectional
Chingwena et al. (2002) [a] [40]	2 years	2934	73	2.49	*Bulinus* spp.	Zimbabwe	Cercarial shedding	Longitudinal
Chingwena et al. (2002) [b] [40]	2 years	2535	1	0.04	*Biomphalaria* spp.	Zimbabwe	Cercarial shedding	Longitudinal
KN de Kock et al. (2004) [29]	Not stated	1639	0	0	*Biomphalaria* spp.	South Africa	Cercarial shedding	Cross-sectional
Donney et al. (1985) [28]	1 year 4 months	3062	62	2.02	*Biomphalaria* spp.	South Africa	Cercarial shedding	Longitudinal
Van Renburg et al. (2016) [a] [20]	2 months	333	0	0	*Bulinus* spp.	Botswana	Cercarial shedding	Longitudinal
Van Renburg et al. (2016) [b] [20]	2 months	325	8	2.46	*Biomphalaria* spp.	Botswana	Cercarial shedding	Longitudinal
Webster et al. (2010) [44]	1 month	1099	42	3.82	*Biomphalaria* spp.	Zimbabwe	Cercarial shedding	Cross-sectional
Wolmarans et al. (2001) [a] [31]	1 year	767	130	16.95	*Bulinus* spp.	South Africa	Cercarial shedding	Longitudinal
Wolmarans et al. (2001) [b] [31]	1 year	932	108	11.59	*Biomphalaria* spp.	South Africa	Cercarial shedding	Longitudinal
Mutsaka-Mukuvaza et al. (2020) [42]	1 year	542	4	0.74	*Biomphalaria* spp.	Zimbabwe	Cercarial shedding	Longitudinal
Madsen et al. (2011) [23]	3 years 10 months		122	0.25	*Bulinus* spp.	Malawi	Cercarial shedding	Longitudinal
Madsen et al. (2011) [1a] [22]	4 years	1970	20	1.02	*Bulinus* spp.	Malawi	Cercarial shedding	Longitudinal
Madsen et al. (2011) [1b] [22]	4 years	6664	22	0.33	*Bulinus* spp.	Malawi	Cercarial shedding	Longitudinal
Chandiwana et al. (1987) [35]	2 years	4452	164	3.68	*Bulinus* spp.	Zimbabwe	Cercarial shedding	Longitudinal
Chandiwana et al. (1988) [2a] [36]	2 years	1851	222	11.99	*Bulinus* spp.	Zimbabwe	Cercarial shedding	Longitudinal
Chandiwana et al. (1988) [2b] [36]	2 years	715	16	2.24	*Biomphalaria* spp.	Zimbabwe	Cercarial shedding	Longitudinal
Chandiwana et al. (1987) [a] [37]	2 years	4452	617	13.86	*Bulinus* spp.	Zimbabwe	Cercarial shedding	Longitudinal
Chandiwana et al. (1987) [b] [37]	2 years	1347	41	3.04	*Biomphalaria* spp.	Zimbabwe	Cercarial shedding	Longitudinal
Chandiwana et al. (1991) [39]	2 months	285	12	4.21	*Bulinus* spp.	Zimbabwe	Cercarial shedding	Cross-sectional
Mungomba et al. (1995) [a] [32]	1 month	135	4	2.96	*Bulinus* spp.	Zambia	Cercarial shedding	Cross-sectional
Mungomba et al. (1995) [a] [32]	1 month	215	17	7.91	*Biomphalaria* spp.	Zambia	Cercarial shedding	Cross-sectional
Madsen et al. (2001) [24]	1 month	992	5	0.50	*Bulinus* spp.	Malawi	Cercarial shedding	Cross-sectional
Poole et al. (2014) [25]	1 month	250	0	0	*Bulinus* spp.	Malawi	Cercarial shedding	Cross-sectional

[a] and [b] represents *Bulinus* spp. and *Biomphalaria* spp. respectively when both species are of interest to the author(s). [1a] and [1b] represents *Bulinus* spp. and *Biomphalaria* spp. respectively when both species are of interest to the author(s) and is the first of two articles published in the same year by the same authors. [2a] and [2b] represents *Bulinus* spp. and *Biomphalaria* spp. respectively when both species are of interest to the author(s) and is the second article published in the same year by the same authors.

## Data Availability

Not applicable.

## Supplementary Materials

The following supporting information can be downloaded at https://www.mdpi.com/article/10.3390/tropicalmed7050072/s1. File S1: Search strategy used to identify selected articles with the search terms; File S2: Quality assessment; Figure S1: Forest plot of subgrouped PPE by countries; Figure S2: Forest plot of subgrouped PPE by snail species. Figure S3: Funnel plot; Figure S4: Figure Doi plot.
