# Supplementary material for: Systematic Review and Meta-Analysis on the Infection Rates of Schistosome Transmitting Snails in Southern Africa
Source: Trop Med Infect Dis. 2022 May 13;7(5):72. doi: 10.3390/tropicalmed7050072 (PMC9145527; doi:10.3390/tropicalmed7050072)
Supplement: Supplementary file 1 [file tropicalmed-07-00072-s001.zip › S1 File Search strategy used to identify selected articles with the search terms.pdf]

Search strategy used to identify selected articles with the search terms, Boolean operators, limits applied on each database and the date the databases were assessed.

**PubMed (12/11/2021)**

39,((((((((((((((Schistosome intermediate host) OR (Intermediate host snails)) OR (Snail intermediate host)) OR (Intermediate host)) OR (freshwater snails)) OR (freshwater snail host)) OR (snail vector)) OR (malacology survey)) OR (malacology survey)) OR (Biomphalaria)) OR (Bulinus)) OR (Bulinid)) AND (((((Infection) OR (Infection rate)) OR (Intensity)) OR (prevalence)) OR (incidence))) AND ((((((Schistosomiasis) OR (Bilharzia)) OR (Bilharziasis)) OR (Schistosoma mansoni)) OR (Schistosoma haematobium)) OR (Schistosoma))) AND ((((((((((Angola) OR (Botswana)) OR (Lesotho)) OR (Mozambique)) OR (Namibia)) OR (South Africa)) OR (Swaziland)) OR (Eswatini)) OR (Zambia)) OR (Zimbabwe)),,,""((("""""schistosoma"""""[MeSH Terms] OR """""schistosoma"""""[All Fields] OR """""schistosome"""""[All Fields] OR """""schistosomes"""""[All Fields] OR """""schistosomal"""""[All Fields]) AND ("""""intermediate"""""[All Fields] OR """""intermediated"""""[All Fields] OR """""intermediately"""""[All Fields] OR """""intermediates"""""[All Fields]) AND """""host"""""[All Fields]) OR (("""""intermediate"""""[All Fields] OR """""intermediated"""""[All Fields] OR """""intermediately"""""[All Fields] OR """""intermediates"""""[All Fields]) AND """""host"""""[All Fields] AND ("""""snail s"""""[All Fields] OR """""snails"""""[MeSH Terms] OR """""snails"""""[All Fields] OR """""snail"""""[All Fields])) OR (("""""snail s"""""[All Fields] OR """""snails"""""[MeSH Terms] OR """""snails"""""[All Fields] OR """""snail"""""[All Fields]) AND ("""""intermediate"""""[All Fields] OR """""intermediated"""""[All Fields] OR """""intermediately"""""[All Fields] OR """""intermediates"""""[All Fields]) AND """""host"""""[All Fields]) OR (("""""intermediate"""""[All Fields] OR """""intermediated"""""[All Fields] OR """""intermediately"""""[All Fields] OR """""intermediates"""""[All Fields]) AND """""host"""""[All Fields]) OR (("""""fresh water"""""[MeSH Terms] OR ("""""fresh"""""[All Fields] AND """""water"""""[All Fields]) OR """""fresh water"""""[All Fields] OR """""freshwater"""""[All Fields] OR """""freshwaters"""""[All Fields]) AND ("""""snail s"""""[All Fields] OR """""snails"""""[MeSH Terms] OR """""snails"""""[All Fields] OR """""snail"""""[All Fields])) OR (("""""fresh water"""""[MeSH Terms] OR ("""""fresh"""""[All Fields] AND """""water"""""[All Fields]) OR """""fresh water"""""[All Fields] OR """""freshwater"""""[All Fields] OR """""freshwaters"""""[All Fields]) AND ("""""snail s"""""[All Fields] OR """""snails"""""[MeSH Terms] OR """""snails"""""[All Fields] OR """""snail"""""[All Fields]) AND """""host"""""[All Fields]) OR (("""""snail s"""""[All Fields] OR """""snails"""""[MeSH Terms] OR """""snails"""""[All Fields] OR """""snail"""""[All Fields]) AND ("""""genetic vectors"""""[MeSH Terms] OR ("""""genetic"""""[All Fields] AND """""vectors"""""[All Fields]) OR """""genetic vectors"""""[All Fields] OR """""vector"""""[All Fields] OR """""disease vectors"""""[MeSH Terms] OR ("""""disease"""""[All Fields] AND """""vectors"""""[All Fields]) OR """""disease vectors"""""[All Fields] OR """""vectors"""""[All Fields] OR """""vector s"""""[All Fields] OR """""vectored"""""[All Fields] OR """""vectoring"""""[All Fields] OR """""vectorization"""""[All Fields] OR """""vectorize"""""[All Fields] OR """""vectorized"""""[All Fields] OR """""vectorizing"""""[All Fields])) OR ("""""malacology"""""[All Fields] AND ("""""survey s"""""[All Fields] OR """""surveyed"""""[All Fields] OR """""surveying"""""[All Fields] OR """""surveys and questionnaires"""""[MeSH Terms] OR ("""""surveys"""""[All Fields] AND

""""questionnaires""""[All Fields]) OR """"surveys and questionnaires""""[All Fields] OR
""""survey""""[All Fields] OR """"surveys""""[All Fields])) OR (""""malacology""""[All
Fields] AND (""""survey s""""[All Fields] OR """"surveyed""""[All Fields] OR
""""surveying""""[All Fields] OR """"surveys and questionnaires""""[MeSH Terms] OR
(""""surveys""""[All Fields] AND """"questionnaires""""[All Fields]) OR """"surveys and
questionnaires""""[All Fields] OR """"survey""""[All Fields] OR """"surveys""""[All
Fields])) OR (""""biomphalaria""""[MeSH Terms] OR """"biomphalaria""""[All Fields]) OR
(""""bulinus""""[MeSH Terms] OR """"bulinus""""[All Fields]) OR """"Bulinid""""[All
Fields]) AND (""""infect""""[All Fields] OR """"infectability""""[All Fields] OR
""""infectable""""[All Fields] OR """"infectant""""[All Fields] OR """"infectants""""[All
Fields] OR """"infected""""[All Fields] OR """"infecteds""""[All Fields] OR
""""infectibility""""[All Fields] OR """"infectible""""[All Fields] OR """"infecting""""[All
Fields] OR """"infection s""""[All Fields] OR """"infections""""[MeSH Terms] OR
""""infections""""[All Fields] OR """"infection""""[All Fields] OR """"infective""""[All
Fields] OR """"infectiveness""""[All Fields] OR """"infectives""""[All Fields] OR
""""infectivities""""[All Fields] OR """"infects""""[All Fields] OR
""""pathogenicity""""[MeSH Subheading] OR """"pathogenicity""""[All Fields] OR
""""infectivity""""[All Fields] OR ((""""infect""""[All Fields] OR """"infectability""""[All
Fields] OR """"infectable""""[All Fields] OR """"infectant""""[All Fields] OR
""""infectants""""[All Fields] OR """"infected""""[All Fields] OR """"infecteds""""[All
Fields] OR """"infectibility""""[All Fields] OR """"infectible""""[All Fields] OR
""""infecting""""[All Fields] OR """"infection s""""[All Fields] OR """"infections""""[MeSH
Terms] OR """"infections""""[All Fields] OR """"infection""""[All Fields] OR
""""infective""""[All Fields] OR """"infectiveness""""[All Fields] OR """"infectives""""[All
Fields] OR """"infectivities""""[All Fields] OR """"infects""""[All Fields] OR
""""pathogenicity""""[MeSH Subheading] OR """"pathogenicity""""[All Fields] OR
""""infectivity""""[All Fields]) AND (""""j rehabil assist technol eng""""[Journal] OR
""""rate""""[All Fields])) OR (""""intense""""[All Fields] OR """"intensely""""[All Fields]
OR """"intensities""""[All Fields] OR """"intensity""""[All Fields] OR
""""intensively""""[All Fields]) OR (""""epidemiology""""[MeSH Subheading] OR
""""epidemiology""""[All Fields] OR """"prevalence""""[All Fields] OR
""""prevalence""""[MeSH Terms] OR """"prevalance""""[All Fields] OR
""""prevalences""""[All Fields] OR """"prevalence s""""[All Fields] OR
""""prevalent""""[All Fields] OR """"prevalently""""[All Fields] OR """"prevalents""""[All
Fields]) OR (""""epidemiology""""[MeSH Subheading] OR """"epidemiology""""[All
Fields] OR """"incidence""""[All Fields] OR """"incidence""""[MeSH Terms] OR
""""incidences""""[All Fields] OR """"incident""""[All Fields] OR """"incidents""""[All
Fields])) AND (""""schistosomiasis""""[MeSH Terms] OR """"schistosomiasis""""[All
Fields] OR """"schistosomiases""""[All Fields] OR (""""schistosomiasis""""[MeSH Terms]
OR """"schistosomiasis""""[All Fields] OR """"bilharzia""""[All Fields] OR
""""schistosoma""""[MeSH Terms] OR """"schistosoma""""[All Fields]) OR
(""""schistosomiasis""""[MeSH Terms] OR """"schistosomiasis""""[All Fields] OR
""""bilharziasis""""[All Fields]) OR (""""schistosoma mansoni""""[MeSH Terms] OR
(""""schistosoma""""[All Fields] AND """"mansoni""""[All Fields]) OR """"schistosoma
mansoni""""[All Fields]) OR (""""schistosoma haematobium""""[MeSH Terms] OR
(""""schistosoma""""[All Fields] AND """"haematobium""""[All Fields]) OR

""""schistosoma haematobium""""[All Fields]) OR (""""schistosoma""""[MeSH Terms] OR """"schistosoma""""[All Fields] OR """"schistosomas""""[All Fields])) AND (""""angola""""[MeSH Terms] OR """"angola""""[All Fields] OR """"angola s""""[All Fields] OR (""""botswana""""[MeSH Terms] OR """"botswana""""[All Fields] OR """"botswana s""""[All Fields]) OR (""""lesotho""""[MeSH Terms] OR """"lesotho""""[All Fields]) OR (""""mozambique""""[MeSH Terms] OR """"mozambique""""[All Fields] OR """"mozambique s""""[All Fields]) OR (""""namibia""""[MeSH Terms] OR """"namibia""""[All Fields]) OR (""""south africa""""[MeSH Terms] OR (""""south""""[All Fields] AND """"africa""""[All Fields]) OR """"south africa""""[All Fields]) OR (""""eswatini""""[MeSH Terms] OR """"eswatini""""[All Fields] OR """"swaziland""""[All Fields]) OR (""""eswatini""""[MeSH Terms] OR """"eswatini""""[All Fields]) OR (""""zambia""""[MeSH Terms] OR """"zambia""""[All Fields] OR """"zambia s""""[All Fields]) OR (""""zimbabwe""""[MeSH Terms] OR """"zimbabwe""""[All Fields] OR """"zimbabwe s""""[All Fields]))"",178,15:39:34"

"38,(((((((((Angola) OR (Botswana)) OR (Lesotho)) OR (Mozambique)) OR (Namibia)) OR (South Africa)) OR (Swaziland)) OR (Eswatini)) OR (Zambia)) OR (Zimbabwe),,,""""""angola""""[MeSH Terms] OR """"angola""""[All Fields] OR """"angola s""""[All Fields] OR (""""botswana""""[MeSH Terms] OR """"botswana""""[All Fields] OR """"botswana s""""[All Fields]) OR (""""lesotho""""[MeSH Terms] OR """"lesotho""""[All Fields]) OR (""""mozambique""""[MeSH Terms] OR """"mozambique""""[All Fields] OR """"mozambique s""""[All Fields]) OR (""""namibia""""[MeSH Terms] OR """"namibia""""[All Fields]) OR (""""south africa""""[MeSH Terms] OR (""""south""""[All Fields] AND """"africa""""[All Fields]) OR """"south africa""""[All Fields]) OR (""""eswatini""""[MeSH Terms] OR """"eswatini""""[All Fields] OR """"swaziland""""[All Fields]) OR (""""eswatini""""[MeSH Terms] OR """"eswatini""""[All Fields]) OR (""""zambia""""[MeSH Terms] OR """"zambia""""[All Fields] OR """"zambia s""""[All Fields]) OR (""""zimbabwe""""[MeSH Terms] OR """"zimbabwe""""[All Fields] OR """"zimbabwe s""""[All Fields])"",""174,704"",15:18:24"

"37,Zimbabwe,,,""""""zimbabwe""""[MeSH Terms] OR """"zimbabwe""""[All Fields] OR """"zimbabwe s""""[All Fields]"",""10,429"",15:16:13"

"36,Zambia,,,""""""zambia""""[MeSH Terms] OR """"zambia""""[All Fields] OR """"zambia s""""[All Fields]"",""8,430"",15:16:03"

"35,Eswatini,,,""""""eswatini""""[MeSH Terms] OR """"eswatini""""[All Fields]"",803,15:15:51"

"34,Swaziland,,,""""""eswatini""""[MeSH Terms] OR """"eswatini""""[All Fields] OR """"swaziland""""[All Fields]"",""1,314"",15:15:41"

"33,South Africa,,,""""""south africa""""[MeSH Terms] OR (""""south""""[All Fields] AND """"africa""""[All Fields]) OR """"south africa""""[All Fields]"",""150,243"",15:15:28"

"32,Namibia,,,""""""namibia""""[MeSH Terms] OR """"namibia""""[All Fields]"",""2,654"",15:15:21"

"31,Mozambique,,,""""""mozambique""""[MeSH Terms] OR """"mozambique""""[All Fields] OR """"mozambique s""""[All Fields]"",""5,336"",15:15:10"

"30,Lesotho,,,""""""lesotho""""[MeSH Terms] OR """"lesotho""""[All Fields]"",931,15:14:57"

"29,Botswana,,,""""""botswana""""[MeSH Terms] OR """"botswana""""[All Fields] OR """"botswana s""""[All Fields]"",""4,078"",15:14:51"

"28,Angola,,,""""""angola""""[MeSH Terms] OR """"angola""""[All Fields] OR """"angola s""""[All Fields]"",""2,012"",15:14:30"

"27,((((((((((((Schistosome intermediate host) OR (Intermediate host snails)) OR (Snail intermediate host)) OR (Intermediate host)) OR (freshwater snails)) OR (freshwater snail host)) OR (snail vector)) OR (malacology survey)) OR (malacology survey)) OR (Biomphalaria)) OR (Bulinus)) OR (Bulinid)) AND (((((Infection) OR (Infection rate)) OR (Intensity)) OR (prevalence)) OR (incidence))) AND ((((((Schistosomiasis) OR (Bilharzia)) OR (Bilharziasis)) OR (Schistosoma mansoni)) OR (Schistosoma haematobium)) OR (Schistosoma)),,,""((((""""schistosoma""""[MeSH Terms] OR """"schistosoma""""[All Fields] OR """"schistosome""""[All Fields] OR """"schistosomes""""[All Fields] OR """"schistosomal""""[All Fields]) AND (""""intermediate""""[All Fields] OR """"intermediated""""[All Fields] OR """"intermediately""""[All Fields] OR """"intermediates""""[All Fields]) AND """"host""""[All Fields]) OR ((""""intermediate""""[All Fields] OR """"intermediated""""[All Fields] OR """"intermediately""""[All Fields] OR """"intermediates""""[All Fields]) AND """"host""""[All Fields] AND (""""snail s""""[All Fields] OR """"snails""""[MeSH Terms] OR """"snails""""[All Fields] OR """"snail""""[All Fields])) OR ((""""snail s""""[All Fields] OR """"snails""""[MeSH Terms] OR """"snails""""[All Fields] OR """"snail""""[All Fields]) AND (""""intermediate""""[All Fields] OR """"intermediated""""[All Fields] OR """"intermediately""""[All Fields] OR """"intermediates""""[All Fields]) AND """"host""""[All Fields]) OR ((""""intermediate""""[All Fields] OR """"intermediated""""[All Fields] OR """"intermediately""""[All Fields] OR """"intermediates""""[All Fields]) AND """"host""""[All Fields]) OR ((""""fresh water""""[MeSH Terms] OR (""""fresh""""[All Fields] AND """"water""""[All Fields]) OR """"fresh water""""[All Fields] OR """"freshwater""""[All Fields] OR """"freshwaters""""[All Fields]) AND (""""snail s""""[All Fields] OR """"snails""""[MeSH Terms] OR """"snails""""[All Fields] OR """"snail""""[All Fields])) OR ((""""fresh water""""[MeSH Terms] OR (""""fresh""""[All Fields] AND """"water""""[All Fields]) OR """"fresh water""""[All Fields] OR """"freshwater""""[All Fields] OR """"freshwaters""""[All Fields]) AND (""""snail s""""[All Fields] OR """"snails""""[MeSH Terms] OR """"snails""""[All Fields] OR """"snail""""[All Fields]) AND """"host""""[All Fields]) OR ((""""snail s""""[All Fields] OR """"snails""""[MeSH Terms] OR """"snails""""[All Fields] OR """"snail""""[All Fields]) AND (""""genetic vectors""""[MeSH Terms] OR (""""genetic""""[All Fields] AND """"vectors""""[All Fields]) OR """"genetic vectors""""[All Fields] OR """"vector""""[All Fields] OR """"disease vectors""""[MeSH Terms] OR (""""disease""""[All Fields] AND """"vectors""""[All Fields]) OR """"disease vectors""""[All Fields] OR """"vectors""""[All Fields] OR """"vector s""""[All Fields] OR """"vectored""""[All Fields] OR """"vectoring""""[All Fields] OR """"vectorization""""[All Fields] OR """"vectorize""""[All Fields] OR """"vectorized""""[All Fields] OR """"vectorizing""""[All Fields])) OR (""""malacology""""[All Fields] AND (""""survey s""""[All Fields] OR """"surveyed""""[All Fields] OR """"surveying""""[All Fields] OR

""""surveys and questionnaires""""[MeSH Terms] OR (""""surveys""""[All Fields] AND """"questionnaires""""[All Fields]) OR """"surveys and questionnaires""""[All Fields] OR """"survey""""[All Fields] OR """"surveys""""[All Fields])) OR (""""malacology""""[All Fields] AND (""""survey s""""[All Fields] OR """"surveyed""""[All Fields] OR """"surveying""""[All Fields] OR """"surveys and questionnaires""""[MeSH Terms] OR (""""surveys""""[All Fields] AND """"questionnaires""""[All Fields]) OR """"surveys and questionnaires""""[All Fields] OR """"survey""""[All Fields] OR """"surveys""""[All Fields])) OR (""""biomphalaria""""[MeSH Terms] OR """"biomphalaria""""[All Fields]) OR (""""bulinus""""[MeSH Terms] OR """"bulinus""""[All Fields]) OR """"Bulinid""""[All Fields]) AND (""""infect""""[All Fields] OR """"infectability""""[All Fields] OR """"infectable""""[All Fields] OR """"infectant""""[All Fields] OR """"infectants""""[All Fields] OR """"infected""""[All Fields] OR """"infecteds""""[All Fields] OR """"infectibility""""[All Fields] OR """"infectible""""[All Fields] OR """"infecting""""[All Fields] OR """"infection s""""[All Fields] OR """"infections""""[MeSH Terms] OR """"infections""""[All Fields] OR """"infection""""[All Fields] OR """"infective""""[All Fields] OR """"infectiveness""""[All Fields] OR """"infectives""""[All Fields] OR """"infectivities""""[All Fields] OR """"infects""""[All Fields] OR """"pathogenicity""""[MeSH Subheading] OR """"pathogenicity""""[All Fields] OR """"infectivity""""[All Fields] OR ((""""infect""""[All Fields] OR """"infectability""""[All Fields] OR """"infectable""""[All Fields] OR """"infectant""""[All Fields] OR """"infectants""""[All Fields] OR """"infected""""[All Fields] OR """"infecteds""""[All Fields] OR """"infectibility""""[All Fields] OR """"infectible""""[All Fields] OR """"infecting""""[All Fields] OR """"infection s""""[All Fields] OR """"infections""""[MeSH Terms] OR """"infections""""[All Fields] OR """"infection""""[All Fields] OR """"infective""""[All Fields] OR """"infectiveness""""[All Fields] OR """"infectives""""[All Fields] OR """"infectivities""""[All Fields] OR """"infects""""[All Fields] OR """"pathogenicity""""[MeSH Subheading] OR """"pathogenicity""""[All Fields] OR """"infectivity""""[All Fields]) AND (""""j rehabil assist technol eng""""[Journal] OR """"rate""""[All Fields])) OR (""""intense""""[All Fields] OR """"intensely""""[All Fields] OR """"intensities""""[All Fields] OR """"intensity""""[All Fields] OR """"intensively""""[All Fields]) OR (""""epidemiology""""[MeSH Subheading] OR """"epidemiology""""[All Fields] OR """"prevalence""""[All Fields] OR """"prevalence""""[MeSH Terms] OR """"prevalance""""[All Fields] OR """"prevalences""""[All Fields] OR """"prevalence s""""[All Fields] OR """"prevalent""""[All Fields] OR """"prevalently""""[All Fields] OR """"prevalents""""[All Fields]) OR (""""epidemiology""""[MeSH Subheading] OR """"epidemiology""""[All Fields] OR """"incidence""""[All Fields] OR """"incidence""""[MeSH Terms] OR """"incidences""""[All Fields] OR """"incident""""[All Fields] OR """"incidents""""[All Fields])) AND (""""schistosomiasis""""[MeSH Terms] OR """"schistosomiasis""""[All Fields] OR """"schistosomiases""""[All Fields] OR (""""schistosomiasis""""[MeSH Terms] OR """"schistosomiasis""""[All Fields] OR """"bilharzia""""[All Fields] OR """"schistosoma""""[MeSH Terms] OR """"schistosoma""""[All Fields]) OR (""""schistosomiasis""""[MeSH Terms] OR """"schistosomiasis""""[All Fields] OR """"bilharziasis""""[All Fields]) OR (""""schistosoma mansoni""""[MeSH Terms] OR (""""schistosoma""""[All Fields] AND """"mansoni""""[All Fields]) OR """"schistosoma mansoni""""[All Fields]) OR (""""schistosoma haematobium""""[MeSH Terms] OR

(""""schistosoma""""[All Fields] AND """"haematobium""""[All Fields]) OR """"schistosoma haematobium""""[All Fields]) OR (""""schistosoma""""[MeSH Terms] OR """"schistosoma""""[All Fields] OR """"schistosomas""""[All Fields]))"",""3,213"",11:23:43"

"26,(((((Schistosomiasis) OR (Bilharzia)) OR (Bilharziasis)) OR (Schistosoma mansoni)) OR (Schistosoma haematobium)) OR (Schistosoma),,,""""""schistosomiasis""""[MeSH Terms] OR """"schistosomiasis""""[All Fields] OR """"schistosomiases""""[All Fields] OR (""""schistosomiasis""""[MeSH Terms] OR """"schistosomiasis""""[All Fields] OR """"bilharzia""""[All Fields] OR """"schistosoma""""[MeSH Terms] OR """"schistosoma""""[All Fields]) OR (""""schistosomiasis""""[MeSH Terms] OR """"schistosomiasis""""[All Fields] OR """"bilharziasis""""[All Fields]) OR (""""schistosoma mansoni""""[MeSH Terms] OR (""""schistosoma""""[All Fields] AND """"mansoni""""[All Fields]) OR """"schistosoma mansoni""""[All Fields]) OR (""""schistosoma haematobium""""[MeSH Terms] OR (""""schistosoma""""[All Fields] AND """"haematobium""""[All Fields]) OR """"schistosoma haematobium""""[All Fields]) OR (""""schistosoma""""[MeSH Terms] OR """"schistosoma""""[All Fields] OR """"schistosomas""""[All Fields])"",""35,447"",11:23:22"

"25,Schistosoma,,,""""""schistosoma""""[MeSH Terms] OR """"schistosoma""""[All Fields] OR """"schistosomas""""[All Fields]"",""23,365"",11:18:59"

"24,Schistosoma haematobium,,,""""""schistosoma haematobium""""[MeSH Terms] OR (""""schistosoma""""[All Fields] AND """"haematobium""""[All Fields]) OR """"schistosoma haematobium""""[All Fields]"",""3,968"",11:18:35"

"23,Schistosoma mansoni,,,""""""schistosoma mansoni""""[MeSH Terms] OR (""""schistosoma""""[All Fields] AND """"mansoni""""[All Fields]) OR """"schistosoma mansoni""""[All Fields]"",""14,546"",11:18:15"

"22,Bilharziasis,,,""""""schistosomiasis""""[MeSH Terms] OR """"schistosomiasis""""[All Fields] OR """"bilharziasis""""[All Fields]"",""28,400"",11:17:59"

"21,Bilharzia,,,""""""schistosomiasis""""[MeSH Terms] OR """"schistosomiasis""""[All Fields] OR """"bilharzia""""[All Fields] OR """"schistosoma""""[MeSH Terms] OR """"schistosoma""""[All Fields]"",""35,248"",11:17:42"

"20,Schistosomiasis,,,""""""schistosomiasis""""[MeSH Terms] OR """"schistosomiasis""""[All Fields] OR """"schistosomiases""""[All Fields]"",""28,197"",11:17:26"

"19,(((((((((((Schistosome intermediate host) OR (Intermediate host snails)) OR (Snail intermediate host)) OR (Intermediate host)) OR (freshwater snails)) OR (freshwater snail host)) OR (snail vector)) OR (malacology survey)) OR (malacology survey)) OR (Biomphalaria)) OR (Bulinus)) OR (Bulinid)) AND (((((Infection) OR (Infection rate)) OR (Intensity)) OR (prevalence)) OR (incidence)),,,""(((""""schistosoma""""[MeSH Terms] OR """"schistosoma""""[All Fields] OR """"schistosome""""[All Fields] OR """"schistosomes""""[All Fields] OR """"schistosomal""""[All Fields]) AND (""""intermediate""""[All Fields] OR """"intermediated""""[All Fields] OR """"intermediately""""[All Fields] OR """"intermediates""""[All Fields]) AND

""""host""""[All Fields]) OR ((""""intermediate""""[All Fields] OR """"intermediated""""[All Fields] OR """"intermediately""""[All Fields] OR """"intermediates""""[All Fields]) AND """"host""""[All Fields] AND (""""snail s""""[All Fields] OR """"snails""""[MeSH Terms] OR """"snails""""[All Fields] OR """"snail""""[All Fields])) OR ((""""snail s""""[All Fields] OR """"snails""""[MeSH Terms] OR """"snails""""[All Fields] OR """"snail""""[All Fields]) AND (""""intermediate""""[All Fields] OR """"intermediated""""[All Fields] OR """"intermediately""""[All Fields] OR """"intermediates""""[All Fields]) AND """"host""""[All Fields]) OR ((""""intermediate""""[All Fields] OR """"intermediated""""[All Fields] OR """"intermediately""""[All Fields] OR """"intermediates""""[All Fields]) AND """"host""""[All Fields]) OR ((""""fresh water""""[MeSH Terms] OR (""""fresh""""[All Fields] AND """"water""""[All Fields]) OR """"fresh water""""[All Fields] OR """"freshwater""""[All Fields] OR """"freshwaters""""[All Fields]) AND (""""snail s""""[All Fields] OR """"snails""""[MeSH Terms] OR """"snails""""[All Fields] OR """"snail""""[All Fields])) OR ((""""fresh water""""[MeSH Terms] OR (""""fresh""""[All Fields] AND """"water""""[All Fields]) OR """"fresh water""""[All Fields] OR """"freshwater""""[All Fields] OR """"freshwaters""""[All Fields]) AND (""""snail s""""[All Fields] OR """"snails""""[MeSH Terms] OR """"snails""""[All Fields] OR """"snail""""[All Fields]) AND """"host""""[All Fields]) OR ((""""snail s""""[All Fields] OR """"snails""""[MeSH Terms] OR """"snails""""[All Fields] OR """"snail""""[All Fields]) AND (""""genetic vectors""""[MeSH Terms] OR (""""genetic""""[All Fields] AND """"vectors""""[All Fields]) OR """"genetic vectors""""[All Fields] OR """"vector""""[All Fields] OR """"disease vectors""""[MeSH Terms] OR (""""disease""""[All Fields] AND """"vectors""""[All Fields]) OR """"disease vectors""""[All Fields] OR """"vectors""""[All Fields] OR """"vector s""""[All Fields] OR """"vectored""""[All Fields] OR """"vectoring""""[All Fields] OR """"vectorization""""[All Fields] OR """"vectorize""""[All Fields] OR """"vectorized""""[All Fields] OR """"vectorizing""""[All Fields])) OR (""""malacology""""[All Fields] AND (""""survey s""""[All Fields] OR """"surveyed""""[All Fields] OR """"surveying""""[All Fields] OR """"surveys and questionnaires""""[MeSH Terms] OR (""""surveys""""[All Fields] AND """"questionnaires""""[All Fields]) OR """"surveys and questionnaires""""[All Fields] OR """"survey""""[All Fields] OR """"surveys""""[All Fields])) OR (""""malacology""""[All Fields] AND (""""survey s""""[All Fields] OR """"surveyed""""[All Fields] OR """"surveying""""[All Fields] OR """"surveys and questionnaires""""[MeSH Terms] OR (""""surveys""""[All Fields] AND """"questionnaires""""[All Fields]) OR """"surveys and questionnaires""""[All Fields] OR """"survey""""[All Fields] OR """"surveys""""[All Fields])) OR (""""biomphalaria""""[MeSH Terms] OR """"biomphalaria""""[All Fields]) OR (""""bulinus""""[MeSH Terms] OR """"bulinus""""[All Fields]) OR """"Bulinid""""[All Fields]) AND (""""infect""""[All Fields] OR """"infectability""""[All Fields] OR """"infectable""""[All Fields] OR """"infectant""""[All Fields] OR """"infectants""""[All Fields] OR """"infected""""[All Fields] OR """"infecteds""""[All Fields] OR """"infectibility""""[All Fields] OR """"infectible""""[All Fields] OR """"infecting""""[All Fields] OR """"infection s""""[All Fields] OR """"infections""""[MeSH Terms] OR """"infections""""[All Fields] OR """"infection""""[All Fields] OR """"infective""""[All Fields] OR """"infectiveness""""[All Fields] OR """"infectives""""[All Fields] OR """"infectivities""""[All Fields] OR """"infects""""[All Fields] OR """"pathogenicity""""[MeSH Subheading] OR

""""pathogenicity""""[All Fields] OR """"infectivity""""[All Fields] OR ((""""infect""""[All Fields] OR """"infectability""""[All Fields] OR """"infectable""""[All Fields] OR """"infectant""""[All Fields] OR """"infectants""""[All Fields] OR """"infected""""[All Fields] OR """"infecteds""""[All Fields] OR """"infectibility""""[All Fields] OR """"infectible""""[All Fields] OR """"infecting""""[All Fields] OR """"infection s""""[All Fields] OR """"infections""""[MeSH Terms] OR """"infections""""[All Fields] OR """"infection""""[All Fields] OR """"infective""""[All Fields] OR """"infectiveness""""[All Fields] OR """"infectives""""[All Fields] OR """"infectivities""""[All Fields] OR """"infects""""[All Fields] OR """"pathogenicity""""[MeSH Subheading] OR """"pathogenicity""""[All Fields] OR """"infectivity""""[All Fields]) AND (""""j rehabil assist technol eng""""[Journal] OR """"rate""""[All Fields])) OR (""""intense""""[All Fields] OR """"intensely""""[All Fields] OR """"intensities""""[All Fields] OR """"intensity""""[All Fields] OR """"intensively""""[All Fields]) OR (""""epidemiology""""[MeSH Subheading] OR """"epidemiology""""[All Fields] OR """"prevalence""""[All Fields] OR """"prevalence""""[MeSH Terms] OR """"prevalance""""[All Fields] OR """"prevalences""""[All Fields] OR """"prevalence s""""[All Fields] OR """"prevalent""""[All Fields] OR """"prevalently""""[All Fields] OR """"prevalents""""[All Fields]) OR (""""epidemiology""""[MeSH Subheading] OR """"epidemiology""""[All Fields] OR """"incidence""""[All Fields] OR """"incidence""""[MeSH Terms] OR """"incidences""""[All Fields] OR """"incident""""[All Fields] OR """"incidents""""[All Fields]))"",""11,056"",11:16:25"

"18,((((Infection) OR (Infection rate)) OR (Intensity)) OR (prevalence)) OR (incidence),,,""""""infect""""[All Fields] OR """"infectability""""[All Fields] OR """"infectable""""[All Fields] OR """"infectant""""[All Fields] OR """"infectants""""[All Fields] OR """"infected""""[All Fields] OR """"infecteds""""[All Fields] OR """"infectibility""""[All Fields] OR """"infectible""""[All Fields] OR """"infecting""""[All Fields] OR """"infection s""""[All Fields] OR """"infections""""[MeSH Terms] OR """"infections""""[All Fields] OR """"infection""""[All Fields] OR """"infective""""[All Fields] OR """"infectiveness""""[All Fields] OR """"infectives""""[All Fields] OR """"infectivities""""[All Fields] OR """"infects""""[All Fields] OR """"pathogenicity""""[MeSH Subheading] OR """"pathogenicity""""[All Fields] OR """"infectivity""""[All Fields] OR ((""""infect""""[All Fields] OR """"infectability""""[All Fields] OR """"infectable""""[All Fields] OR """"infectant""""[All Fields] OR """"infectants""""[All Fields] OR """"infected""""[All Fields] OR """"infecteds""""[All Fields] OR """"infectibility""""[All Fields] OR """"infectible""""[All Fields] OR """"infecting""""[All Fields] OR """"infection s""""[All Fields] OR """"infections""""[MeSH Terms] OR """"infections""""[All Fields] OR """"infection""""[All Fields] OR """"infective""""[All Fields] OR """"infectiveness""""[All Fields] OR """"infectives""""[All Fields] OR """"infectivities""""[All Fields] OR """"infects""""[All Fields] OR """"pathogenicity""""[MeSH Subheading] OR """"pathogenicity""""[All Fields] OR """"infectivity""""[All Fields]) AND (""""j rehabil assist technol eng""""[Journal] OR """"rate""""[All Fields])) OR (""""intense""""[All Fields] OR """"intensely""""[All Fields] OR """"intensities""""[All Fields] OR """"intensity""""[All Fields] OR """"intensively""""[All Fields]) OR (""""epidemiology""""[MeSH Subheading] OR """"epidemiology""""[All Fields] OR """"prevalence""""[All Fields] OR """"prevalence""""[MeSH Terms] OR """"prevalance""""[All Fields] OR

""""prevalences""""[All Fields] OR """"prevalence s""""[All Fields] OR """"prevalent""""[All Fields] OR """"prevalently""""[All Fields] OR """"prevalents""""[All Fields]) OR (""""epidemiology""""[MeSH Subheading] OR """"epidemiology""""[All Fields] OR """"incidence""""[All Fields] OR """"incidence""""[MeSH Terms] OR """"incidences""""[All Fields] OR """"incident""""[All Fields] OR """"incidents""""[All Fields])"",""6,825,276"",11:16:03"

"17,incidence,,,""""""epidemiology""""[MeSH Subheading] OR """"epidemiology""""[All Fields] OR """"incidence""""[All Fields] OR """"incidence""""[MeSH Terms] OR """"incidences""""[All Fields] OR """"incident""""[All Fields] OR """"incidents""""[All Fields]"",""3,234,667"",11:15:24"

"16,prevalence,,,""""""epidemiology""""[MeSH Subheading] OR """"epidemiology""""[All Fields] OR """"prevalence""""[All Fields] OR """"prevalence""""[MeSH Terms] OR """"prevalance""""[All Fields] OR """"prevalences""""[All Fields] OR """"prevalence s""""[All Fields] OR """"prevalent""""[All Fields] OR """"prevalently""""[All Fields] OR """"prevalents""""[All Fields]"",""3,103,946"",11:15:11"

"15,Intensity,,,""""""intense""""[All Fields] OR """"intensely""""[All Fields] OR """"intensities""""[All Fields] OR """"intensity""""[All Fields] OR """"intensively""""[All Fields]"",""559,177"",11:14:51"

"14,Infection rate,,,""(""""infect""""[All Fields] OR """"infectability""""[All Fields] OR """"infectable""""[All Fields] OR """"infectant""""[All Fields] OR """"infectants""""[All Fields] OR """"infected""""[All Fields] OR """"infecteds""""[All Fields] OR """"infectibility""""[All Fields] OR """"infectible""""[All Fields] OR """"infecting""""[All Fields] OR """"infection s""""[All Fields] OR """"infections""""[MeSH Terms] OR """"infections""""[All Fields] OR """"infection""""[All Fields] OR """"infective""""[All Fields] OR """"infectiveness""""[All Fields] OR """"infectives""""[All Fields] OR """"infectivities""""[All Fields] OR """"infects""""[All Fields] OR """"pathogenicity""""[MeSH Subheading] OR """"pathogenicity""""[All Fields] OR """"infectivity""""[All Fields]) AND (""""j rehabil assist technol eng""""[Journal] OR """"rate""""[All Fields])"",""289,492"",11:14:34"

"13,Infection,,,""""""infect""""[All Fields] OR """"infectability""""[All Fields] OR """"infectable""""[All Fields] OR """"infectant""""[All Fields] OR """"infectants""""[All Fields] OR """"infected""""[All Fields] OR """"infecteds""""[All Fields] OR """"infectibility""""[All Fields] OR """"infectible""""[All Fields] OR """"infecting""""[All Fields] OR """"infection s""""[All Fields] OR """"infections""""[MeSH Terms] OR """"infections""""[All Fields] OR """"infection""""[All Fields] OR """"infective""""[All Fields] OR """"infectiveness""""[All Fields] OR """"infectives""""[All Fields] OR """"infectivities""""[All Fields] OR """"infects""""[All Fields] OR """"pathogenicity""""[MeSH Subheading] OR """"pathogenicity""""[All Fields] OR """"infectivity""""[All Fields]"",""3,649,597"",11:14:28"

"12,((((((((((Schistosome intermediate host) OR (Intermediate host snails)) OR (Snail intermediate host)) OR (Intermediate host)) OR (freshwater snails)) OR (freshwater snail host)) OR (snail vector)) OR (malacology survey)) OR (malacology survey)) OR (Biomphalaria)) OR (Bulinus)) OR (Bulinid),,,""((""""schistosoma""""[MeSH Terms] OR

""""schistosoma""""[All Fields] OR """"schistosome""""[All Fields] OR """"schistosomes""""[All Fields] OR """"schistosomal""""[All Fields]) AND (""""intermediate""""[All Fields] OR """"intermediated""""[All Fields] OR """"intermediately""""[All Fields] OR """"intermediates""""[All Fields]) AND """"host""""[All Fields]) OR ((""""intermediate""""[All Fields] OR """"intermediated""""[All Fields] OR """"intermediately""""[All Fields] OR """"intermediates""""[All Fields]) AND """"host""""[All Fields] AND (""""snail s""""[All Fields] OR """"snails""""[MeSH Terms] OR """"snails""""[All Fields] OR """"snail""""[All Fields])) OR ((""""snail s""""[All Fields] OR """"snails""""[MeSH Terms] OR """"snails""""[All Fields] OR """"snail""""[All Fields]) AND (""""intermediate""""[All Fields] OR """"intermediated""""[All Fields] OR """"intermediately""""[All Fields] OR """"intermediates""""[All Fields]) AND """"host""""[All Fields]) OR ((""""intermediate""""[All Fields] OR """"intermediated""""[All Fields] OR """"intermediately""""[All Fields] OR """"intermediates""""[All Fields]) AND """"host""""[All Fields]) OR ((""""fresh water""""[MeSH Terms] OR (""""fresh""""[All Fields] AND """"water""""[All Fields]) OR """"fresh water""""[All Fields] OR """"freshwater""""[All Fields] OR """"freshwaters""""[All Fields]) AND (""""snail s""""[All Fields] OR """"snails""""[MeSH Terms] OR """"snails""""[All Fields] OR """"snail""""[All Fields])) OR ((""""fresh water""""[MeSH Terms] OR (""""fresh""""[All Fields] AND """"water""""[All Fields]) OR """"fresh water""""[All Fields] OR """"freshwater""""[All Fields] OR """"freshwaters""""[All Fields]) AND (""""snail s""""[All Fields] OR """"snails""""[MeSH Terms] OR """"snails""""[All Fields] OR """"snail""""[All Fields]) AND """"host""""[All Fields]) OR ((""""snail s""""[All Fields] OR """"snails""""[MeSH Terms] OR """"snails""""[All Fields] OR """"snail""""[All Fields]) AND (""""genetic vectors""""[MeSH Terms] OR (""""genetic""""[All Fields] AND """"vectors""""[All Fields]) OR """"genetic vectors""""[All Fields] OR """"vector""""[All Fields] OR """"disease vectors""""[MeSH Terms] OR (""""disease""""[All Fields] AND """"vectors""""[All Fields]) OR """"disease vectors""""[All Fields] OR """"vectors""""[All Fields] OR """"vector s""""[All Fields] OR """"vectored""""[All Fields] OR """"vectoring""""[All Fields] OR """"vectorization""""[All Fields] OR """"vectorize""""[All Fields] OR """"vectorized""""[All Fields] OR """"vectorizing""""[All Fields])) OR (""""malacology""""[All Fields] AND (""""survey s""""[All Fields] OR """"surveyed""""[All Fields] OR """"surveying""""[All Fields] OR """"surveys and questionnaires""""[MeSH Terms] OR (""""surveys""""[All Fields] AND """"questionnaires""""[All Fields]) OR """"surveys and questionnaires""""[All Fields] OR """"survey""""[All Fields] OR """"surveys""""[All Fields])) OR (""""malacology""""[All Fields] AND (""""survey s""""[All Fields] OR """"surveyed""""[All Fields] OR """"surveying""""[All Fields] OR """"surveys and questionnaires""""[MeSH Terms] OR (""""surveys""""[All Fields] AND """"questionnaires""""[All Fields]) OR """"surveys and questionnaires""""[All Fields] OR """"survey""""[All Fields] OR """"surveys""""[All Fields])) OR (""""biomphalaria""""[MeSH Terms] OR """"biomphalaria""""[All Fields]) OR (""""bulinus""""[MeSH Terms] OR """"bulinus""""[All Fields]) OR """"Bulinid""""[All Fields]"",""18,609"",11:12:05"

"11,Bulinid,,,""""""Bulinid""""[All Fields]"",31,11:07:11"

"10,Bulinus,,,""""""bulinus""""[MeSH Terms] OR """"bulinus""""[All Fields]"",959,11:06:51"

"9,Biomphalaria,,,""""""biomphalaria""""[MeSH Terms] OR """"biomphalaria""""[All Fields]"",""3,313"",11:06:37"

"8,malacology survey,,,""""""malacology""""[All Fields] AND (""""survey s""""[All Fields] OR """"surveyed""""[All Fields] OR """"surveying""""[All Fields] OR """"surveys and questionnaires""""[MeSH Terms] OR (""""surveys""""[All Fields] AND """"questionnaires""""[All Fields]) OR """"surveys and questionnaires""""[All Fields] OR """"survey""""[All Fields] OR """"surveys""""[All Fields])"",25,11:06:14"

"7,snail vector,,,""(""""snail s""""[All Fields] OR """"snails""""[MeSH Terms] OR """"snails""""[All Fields] OR """"snail""""[All Fields]) AND (""""genetic vectors""""[MeSH Terms] OR (""""genetic""""[All Fields] AND """"vectors""""[All Fields]) OR """"genetic vectors""""[All Fields] OR """"vector""""[All Fields] OR """"disease vectors""""[MeSH Terms] OR (""""disease""""[All Fields] AND """"vectors""""[All Fields]) OR """"disease vectors""""[All Fields] OR """"vectors""""[All Fields] OR """"vector s""""[All Fields] OR """"vectored""""[All Fields] OR """"vectoring""""[All Fields] OR """"vectorization""""[All Fields] OR """"vectorize""""[All Fields] OR """"vectorized""""[All Fields] OR """"vectorizing""""[All Fields])"",""2,316"",11:05:50"

"6,freshwater snail host,,,""(""""fresh water""""[MeSH Terms] OR (""""fresh""""[All Fields] AND """"water""""[All Fields]) OR """"fresh water""""[All Fields] OR """"freshwater""""[All Fields] OR """"freshwaters""""[All Fields]) AND (""""snail s""""[All Fields] OR """"snails""""[MeSH Terms] OR """"snails""""[All Fields] OR """"snail""""[All Fields]) AND """"host""""[All Fields]"",738,11:05:32"

"5,freshwater snails,,,""(""""fresh water""""[MeSH Terms] OR (""""fresh""""[All Fields] AND """"water""""[All Fields]) OR """"fresh water""""[All Fields] OR """"freshwater""""[All Fields] OR """"freshwaters""""[All Fields]) AND (""""snail s""""[All Fields] OR """"snails""""[MeSH Terms] OR """"snails""""[All Fields] OR """"snail""""[All Fields])"",""3,233"",11:05:19"

"4,Intermediate host,,,""(""""intermediate""""[All Fields] OR """"intermediated""""[All Fields] OR """"intermediately""""[All Fields] OR """"intermediates""""[All Fields]) AND """"host""""[All Fields]"",""12,120"",11:05:01"

"3,Snail intermediate host,,,""(""""snail s""""[All Fields] OR """"snails""""[MeSH Terms] OR """"snails""""[All Fields] OR """"snail""""[All Fields]) AND (""""intermediate""""[All Fields] OR """"intermediated""""[All Fields] OR """"intermediately""""[All Fields] OR """"intermediates""""[All Fields]) AND """"host""""[All Fields]"",""2,021"",11:04:45"

"2,Intermediate host snails,,,""(""""intermediate""""[All Fields] OR """"intermediated""""[All Fields] OR """"intermediately""""[All Fields] OR """"intermediates""""[All Fields]) AND """"host""""[All Fields] AND (""""snail s""""[All Fields] OR """"snails""""[MeSH Terms] OR """"snails""""[All Fields] OR """"snail""""[All Fields])"",""2,021"",11:04:18"

"1,Schistosome intermediate host,,,""(""""schistosoma""""[MeSH Terms] OR """"schistosoma""""[All Fields] OR """"schistosome""""[All Fields] OR """"schistosomes""""[All Fields] OR """"schistosomal""""[All Fields]) AND (""""intermediate""""[All Fields] OR """"intermediated""""[All Fields] OR

"""""intermediately"""""[All Fields] OR """""intermediates"""""[All Fields]) AND """""host"""""[All Fields]"",""1,084"",11:03:05"
