# Supplementary material for: Systematic Review and Meta-Analysis on the Infection Rates of Schistosome Transmitting Snails in Southern Africa
Source: Trop Med Infect Dis. 2022 May 13;7(5):72. doi: 10.3390/tropicalmed7050072 (PMC9145527; doi:10.3390/tropicalmed7050072)
Supplement: Supplementary file 1 [file tropicalmed-07-00072-s001.zip › S2 Figure Forest plot of subgrouped PPE by snail species.pdf]

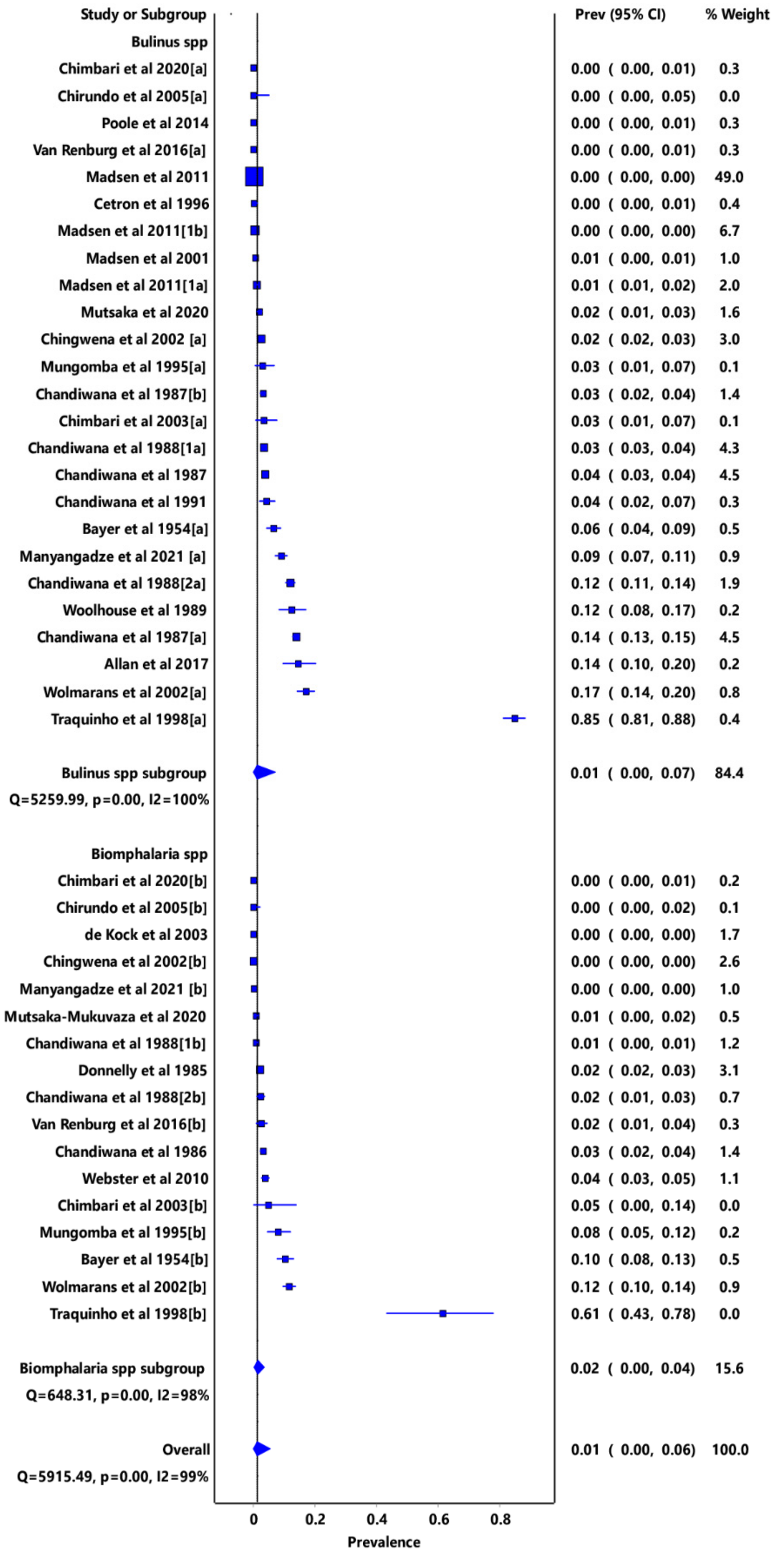

Figure S2 Forest plot of subgrouped PPE by snail species. [a] and [b] represents *Bulinus spp* and *Biomphalaria spp* respectively when both species are of interest to the author(s). [1a] and [1b] represents *Bulinus spp* and *Biomphalaria spp* respectively when both species are of interest to the author(s) and is the first of two articles published in the same year by the same authors. [2a] and [2b] represents *Bulinus spp* and *Biomphalaria spp* respectively when both species are of interest to the author(s) and is the second article published in the same year.
