# Supplementary material for: Systematic Review and Meta-Analysis on the Infection Rates of Schistosome Transmitting Snails in Southern Africa
Source: Trop Med Infect Dis. 2022 May 13;7(5):72. doi: 10.3390/tropicalmed7050072 (PMC9145527; doi:10.3390/tropicalmed7050072)
Supplement: Supplementary file 1 [file tropicalmed-07-00072-s001.zip › S2 File Quality assessment.pdf]

Quality assessment of the articles included.

The Joanna Briggs Institute Critical Appraisal Tool for studies reporting prevalence data was used to assess the quality of all studies included in the review. The following questions were accessed using the tool assessed the about the articles:

i. sample representative of the target population,
ii. study participants recruited in an appropriate way,
iii. sample size adequate
iv. study subjects and setting described in detail
v. data analysis conducted with sufficient coverage of the identified sample
vi. objective standard criteria used for measurement of the condition
vii. condition measured reliably,
viii. appropriate statistical analysis,
ix. important confounding factors/ subgroups/differences identified and accounted for,
x. subpopulations identified using objective criteria.

First Author and Year	Was the sample representative of the target population?	Were study participants recruited in an appropriate way?	Was the sample size adequate?	Were the study subjects and setting described in detail?	Is the data analysis conducted with sufficient coverage of the identified sample?	Were objective, standard criteria used for measurement of the condition?	Was the condition measured reliably?	Was there appropriate statistical analysis?	Are all important confounding factors/ subgroups/differences identified and accounted for?	Were subpopulations identified using objective criteria?	Quality score
Allan et al. 2017	Yes	Yes	Yes	Yes	Yes	Yes	Yes	Yes	No	Yes	9
Bayer et al. 1954	Yes	Yes	Yes	Yes	Yes	Yes	Yes	Yes	No	Yes	9
Cetron et al. 1996	Yes	Yes	Yes	Yes	Yes	Yes	Yes	Yes	No	Yes	9
Chandiwana et al. 1986	Yes	Yes	Yes	Yes	Yes	Yes	Yes	Yes	No	Yes	9
Chandiwana et al. 1987	Yes	Yes	Yes	Yes	Yes	Yes	Yes	Yes	Yes	Yes	10
Chandiwana et al. 1987 [a]	Yes	Yes	Yes	Yes	Yes	Yes	Yes	Yes	Yes	Yes	10
Chandiwana et al. 1988	Yes	Yes	Yes	Yes	Yes	Yes	Yes	Yes	No	Yes	9
Chandiwana et al. 1988 [2a]	Yes	Yes	Yes	Yes	Yes	Yes	Yes	Yes	Yes	Yes	10
Chandiwana et al. 1991	Yes	Yes	Yes	Yes	Yes	Yes	Yes	Yes	Yes	Yes	10
Chimbari et al. 2003	Yes	Yes	Yes	Yes	Yes	Yes	Yes	Yes	No	Yes	9
Chimbari et al. 2020	Yes	Yes	Yes	Yes	Yes	Yes	Yes	Yes	Yes	Yes	10
Chingwena et al. 2002	Yes	Yes	Yes	Yes	Yes	Yes	Yes	Yes	No	Yes	9
Chirundo et al. 2005	Yes	Yes	Yes	Yes	Yes	Yes	Yes	Yes	No	Yes	9
De KN Kock et al. 2003	Yes	Yes	Yes	Yes	Yes	Yes	Yes	Yes	Yes	Yes	10
Donney et al. 1985	Yes	Yes	Yes	Yes	Yes	Yes	Yes	Yes	Yes	Yes	10
Lukezo et al. 1995	Yes	Yes	Yes	Yes	Yes	Yes	Yes	Yes	No	Yes	9
Madsen et al. 2001	Yes	Yes	Yes	Yes	Yes	Yes	Yes	Yes	No	Yes	9
Madsen et al. 2011 (Density)	Yes	Yes	Yes	Yes	Yes	Yes	Yes	Yes	No	Yes	9
Madsen et al. 2011[a]	Yes	Yes	Yes	Yes	Yes	Yes	Yes	Yes	No	Yes	9
Manyangadze et al. 2021	Yes	Yes	Yes	Yes	Yes	Yes	Yes	Yes	Yes	Yes	10
Mutsaka et al. 2020	Yes	Yes	Yes	Yes	Yes	Yes	Yes	Yes	No	Yes	9
Mutsaka-Mukuvaza et al. 2020	Yes	Yes	Yes	Yes	Yes	Yes	Yes	Yes	No	Yes	9
Poole et al 2014	Yes	Yes	Yes	Yes	Yes	Yes	Yes	Yes	No	Yes	9

Traquinho et al. 1998	Yes	Yes	Yes	Yes	Yes	Yes	Yes	Yes	No	Yes	9
Van Renburg et al. 2016	Yes	Yes	Yes	Yes	Yes	Yes	Yes	Yes	No	Yes	9
Webster et al. 2010	Yes	Yes	Yes	Yes	Yes	Yes	Yes	Yes	Yes	Yes	10
Wolmarans et al. 2001	Yes	Yes	Yes	Yes	Yes	Yes	Yes	Yes	Yes	Yes	10
Woolhouse et al. 1989	Yes	Yes	Yes	Yes	Yes	Yes	Yes	Yes	NA	Yes	9
