# Supplementary material for: Systematic Review and Meta-Analysis on the Infection Rates of Schistosome Transmitting Snails in Southern Africa
Source: Trop Med Infect Dis. 2022 May 13;7(5):72. doi: 10.3390/tropicalmed7050072 (PMC9145527; doi:10.3390/tropicalmed7050072)
Supplement: Supplementary file 1 [file tropicalmed-07-00072-s001.zip › S3 Figure Funnel Plot.pdf]

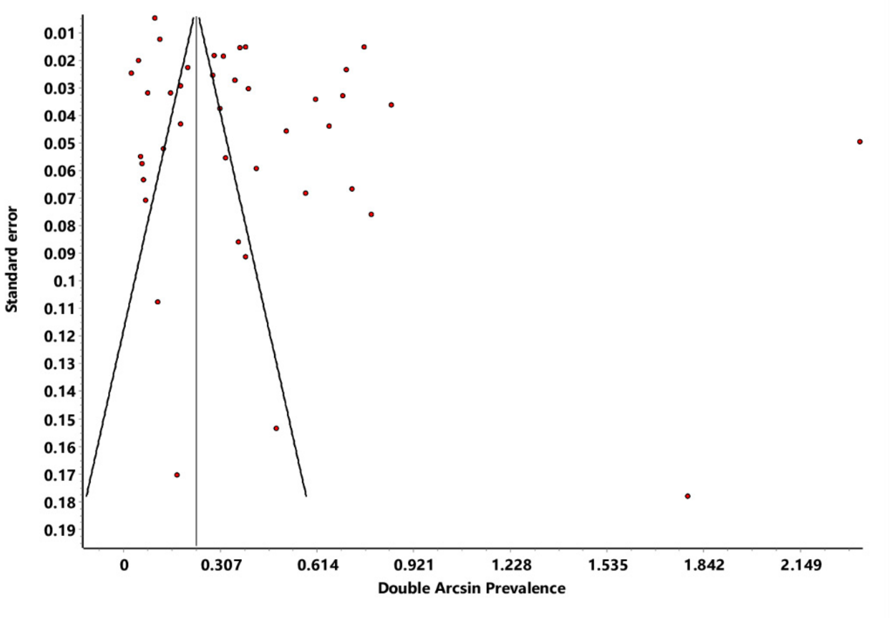

Figure S3: Funnel plot of the double arcsine prevalence estimates infection rates of Schistosome Transmitting Snails in Southern Africa.
